# Temporal trends in mental health terminology in Alzheimer’s disease clinical trials

**DOI:** 10.1371/journal.pone.0310264

**Published:** 2024-12-30

**Authors:** Amir-Ali Golrokhian-Sani, Maya Morcos, Alecco Philippi, Reem Al-Rawi, Marc Morcos, Rui Fu

**Affiliations:** 1 Faculty of Medicine, University of Ottawa, Ottawa, Ontario, Canada; 2 Faculty of Health Sciences, Queen’s University, Kingston, Ontario, Canada; 3 Faculty of Applied Science and Engineering, University of Toronto, Toronto, Ontario, Canada; 4 Department of Otolaryngology-Head & Neck Surgery, Sunnybrook Health Sciences Centre, Toronto, Ontario, Canada; Federal University of Paraiba, BRAZIL

## Abstract

**Background:**

Despite several studies having correlated Alzheimer’s disease with mental health conditions, the extent to which they have been incorporated into Alzheimer’s disease clinical trials remains unclear.

**Objective:**

This study aimed to assess the temporal trends in mental health-related terminology in Alzheimer’s disease clinical trials as a proxy measure of research interest. Additionally, it sought to determine the effect of the COVID-19 pandemic on the frequency of these terms through pre-pandemic and post-pandemic trend assessment.

**Methods:**

In this retrospective descriptive analysis, we included 2243 trials with a start date between 1988 and 2022 by searching for the keyword “Alzheimer Disease” in the U.S. National Library of Medicine ClinicaTrials.gov database. A Python program was created to extract and count the frequency of four mental health terms (loneliness, depression, anxiety, and distress) by year and trial status (e.g., completed, active, recruiting). Binary logistic regression analyses were conducted to examine the yearly patterns in the appearance of the four mental health terms. A multivariable logistic regression analysis was performed to identify trial characteristics associated with each mental health term.

**Results:**

Our results depicted a statistically significant increasing trend in three (i.e., loneliness, anxiety, distress) of the four mental health conditions by year. A comparison between pre-pandemic and post-pandemic trials showed an increase in the mention of the same three words over time.

**Interpretation:**

These results may suggest a growing awareness of mental health conditions and a greater interest in considering these conditions in Alzheimer’s disease trials, particularly after the onset of COVID-19. Future researchers should conduct more in-depth analyses to examine how mental health variables are operationalized in these trials, with consideration for their subsequent success.

## Introduction

### Background

As Alzheimer’s disease (AD) research rapidly advances, it becomes increasingly crucial to understand its intersection with mental health. Dementia is a term used to describe a group of cognitive impairments involving a decline in thinking capacity, memory, and independence [1]. AD, the most prevalent form of dementia, constitutes 60–70% of cases, with its prevalence skyrocketing by 160% from 1990 to 2019 [1, 2]. This increase underscores the urgent need for effective management strategies, as current treatments predominantly address symptoms yet do not halt disease progression [3]. Dementia presents profound challenges not only to those diagnosed but also to their caregivers. Individuals with dementia often face a progressive decline in cognitive functions and daily living skills, which can lead to significant emotional distress and reduced quality of life [4, 5]. Caregivers, in turn, frequently experience high levels of stress and burnout due to the constant demands of providing care and emotional support [6]. Various risk factors of AD manifestation have been established in the literature, including female sex, advancing age, traumatic brain injury, smoking, and obesity [7]. In addition, mental health (MH) conditions, such as anxiety, depression, psychological distress, and loneliness, have been found to elevate the risk of AD [7–9].

### Addressing the gaps

Over the past few decades, MH conditions have been gradually destigmatized, resulting in a steady increase in pertinent studies [10]. However, it is unclear if similar trends exist in AD research. Given the goal of managing both diseases concurrently, it is imperative to include MH variables in AD trials to refine the current treatment protocol. As such, understanding the temporal trends may guide prospective researchers in identifying gaps in knowledge and inform their future research. This information may also help set research priorities, which can guide resource allocation to support the development of treatments. Such treatments can help target both AD and MH or alleviate certain MH conditions in patients already affected by AD (such as cognitive behavioral therapy) [11].

### Rationale and objectives

Currently, no study has quantified MH-related research interest in AD trials. The objectives of this study include: 1) to develop a Python program that can extract and capture the frequency of MH terms in AD trial summaries as a proxy measure for research interest, and 2) to test if such MH research interest among AD trial researchers has changed with time and through the COVID-19 pandemic. We hypothesize an increase in the frequency of MH terminology in AD trials in more recent years, given the destigmatization of MH conditions and the peaked interest in these conditions following the COVID-19 pandemic.

## Methods

### Study design

**Fig 1:**
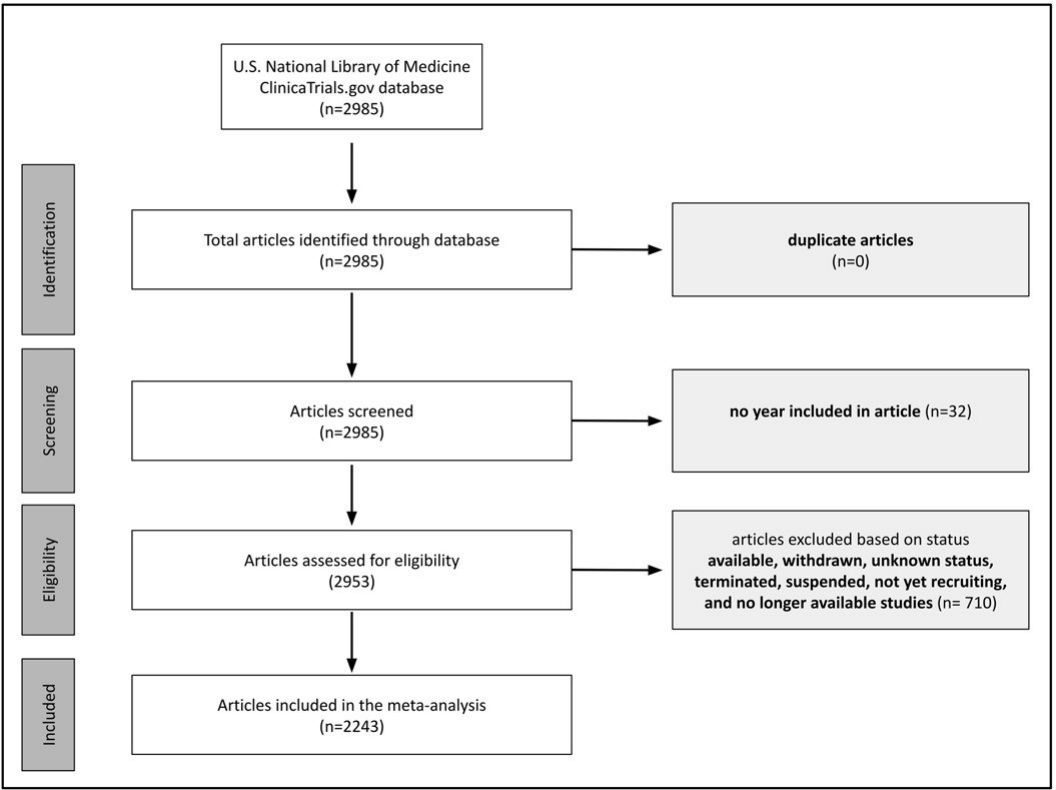
Flow chart outlining the article screening process.

This retrospective descriptive study was conducted in November 2022 using the U.S. National Library of Medicine ClinicalTrials.gov database. No patient medical files were accessed. We extracted the abstracts of trial studies with a start date between 1988 and 2022 utilizing a keyword search of “Alzheimer Disease” on the website. Trials with the following statuses were excluded: available (this database category had missing dates), withdrawn, unknown status, terminated, suspended, not yet recruiting, and no longer available (Fig 1). Studies that were recruiting, enrolling by invitation, completed, and active not recruiting were included. For the included studies, their XML files and an Excel sheet listing their starting dates and national clinical trial (NCT) numbers were downloaded. A Python program was written to process each XML file and extract the brief and detailed descriptions. The program removed punctuation by filtering out any symbol other than words, numbers, whitespace, apostrophes, and dashes. Symbols removed were replaced with whitespace, and all text was converted to lowercase. Following this, all text was split into a list of words, and any instances of an apostrophe followed by an “s” (i.e., ‘s) at the end of a word were removed (to account for differences such as “Alzheimer” versus “Alzheimer’s”).

### Outcome

In this analysis, we focused on four MH conditions that have a common coexistence with AD in prior literature: depression, anxiety, distress, and loneliness [12–15]. For instance, depression is recognized as one of AD’s core symptoms, with late-life depression doubling AD risk [12]. Sutin et al. found a 40% increased risk of dementia in lonely people [16]. Zhao et al. found that the prevalence of anxiety across 25 AD studies ranges from 12–70%, making it a major factor [17]. Finally, Wilson et al. found that individuals who were distress-prone were 2.7 times more likely to develop AD in roughly the next 3 years [18]. There is no denying that there are other terms related to MH in AD research that could have also been assessed, but these four form some of the most commonly associated terms and provide a strong foundation for testing our code. The primary outcomes were the frequency of these MH terms in AD trial documents as a proxy to quantify the research interest of studying these conditions. For each included AD trial, we calculated the frequency of each of the four MH conditions as the number of times the word appeared divided by the total word count in the brief and detailed descriptions. This would take into account the relative value of the word compared to the paragraph in which it is found. In summary, our outcomes were based on whether the MH terms in question were mentioned in the description sections of clinical trials in our database. Number of mentions, frequency, and a binary yes/no for mentions were all collected.

### Statistical analysis

For trials of each status, we used Pearson’s correlation coefficient (r) to assess the strength of the relationship between the frequency of each MH condition and the trial start date (year) using GraphPad Prism 9. Python was utilized to extract the data and organize it in a manner suitable to our statistical software. The following analyses were conducted utilizing IBM SPSS version 29 and Microsoft Excel version 2401. For each MH term, we conducted a multivariable logistic regression analysis to evaluate the odds of including this MH term using COVID-19 period (COVID-19 vs. pre-COVID) and trial completion (1 = completed status, 0 = not completed) as independent variables. The year 2020 was used to indicate the start of the large-scale global pandemic control measures [19, 20]. We repeated this logistic regression analysis to assess the odds of including at least one of the four MH terms. Additionally, binary logistic regressions were performed examining the association between start year and whether the MH terminology was mentioned (1 = yes, 0 = no) for all four terms individually and together. This analysis was selected due to its robustness and appropriateness for the outcome being evaluated. Throughout the code, 1 and 0 represent their standard binary values of true and false, respectively. Additionally, sensitivity and specificity analyses were performed to determine the more impactful variables. They were complemented by a check for multicollinearity to discover whether any variables were inflating the model.

All analyses were 2-sided, where p-value<0.05 was set to indicate statistical significance. The Python code developed for data sorting, as well as the exported data, can be accessed at https://zenodo.org/records/11052250.

## Results

**Fig 2:**
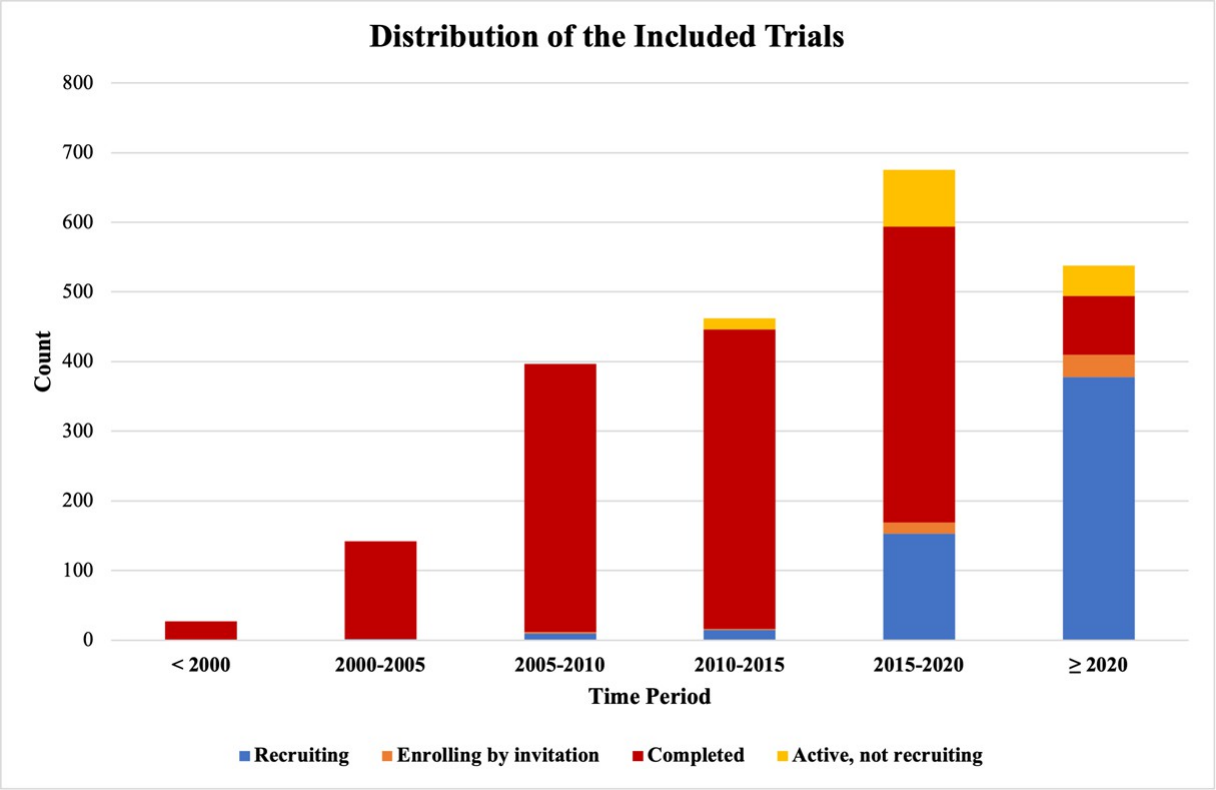
Volume of trials by current status and start year.

The multivariable regression included 2243 studies (Fig 1). The majority of these trials were in the “completed” category (n = 1492, 66.6%). The number of studies generally increased with time in all four statuses (Fig 2). Trials that started in the COVID-19 period were associated with noticeably improved odds of containing one of the four MH terms (excluding “depression”) compared to their pre-COVID counterparts (Table 1). Furthermore, trials in the “completed” category were associated with 5.850 and 2.760 higher odds of containing “loneliness” and “distress,” respectively.

**Table 1:** Multivariable logistic regression analysis on the effects of COVID-19 and trial completion on the mention (y/n) of MH terminology (n = 2243).

	B	Standard Error	P-value	Exp(B)*	Lower 95% for Exp(B)	Upper 95% for (Exp(B)	Log Likelihood	Likelihood Ratio Significance
Depression (y/n)								
COVID? (y/n)	0.068	0.235	0.773	1.070	0.675	1.696	23.129	0.773
Complete? (y/n)	-0.187	0.215	0.386	0.830	0.544	1.266	23.785	0.390
Anxiety (y/n)								
COVID? (y/n)	1.134	0.338	<0.001	3.109	1.604	6.024	30.610	<0.001
Complete? (y/n)	0.365	0.339	0.281	1.440	0.742	2.798	20.623	0.278
Loneliness (y/n)								
COVID? (y/n)	3.846	0.833	<0.001	46.810	9.146	239.583	35.450	<0.001
Complete? (y/n)	1.766	0.635	0.005	5.850	1.684	20.323	16.841	0.007
Distress (y/n)								
COVID? (y/n)	1.344	0.388	<0.001	3.835	1.794	8.198	27.667	<0.001
Complete? (y/n)	1.015	0.409	0.013	2.760	1.238	6.153	22.970	0.012
Any Word (y/n)								
COVID? (y/n)	0.543	0.205	0.008	1.722	1.152	2.573	40.145	0.008
Complete? (y/n)	0.116	0.197	0.556	1.123	0.763	1.652	33.510	0.554

*Exp(B) represents the odds ratio.

**Fig 3:**
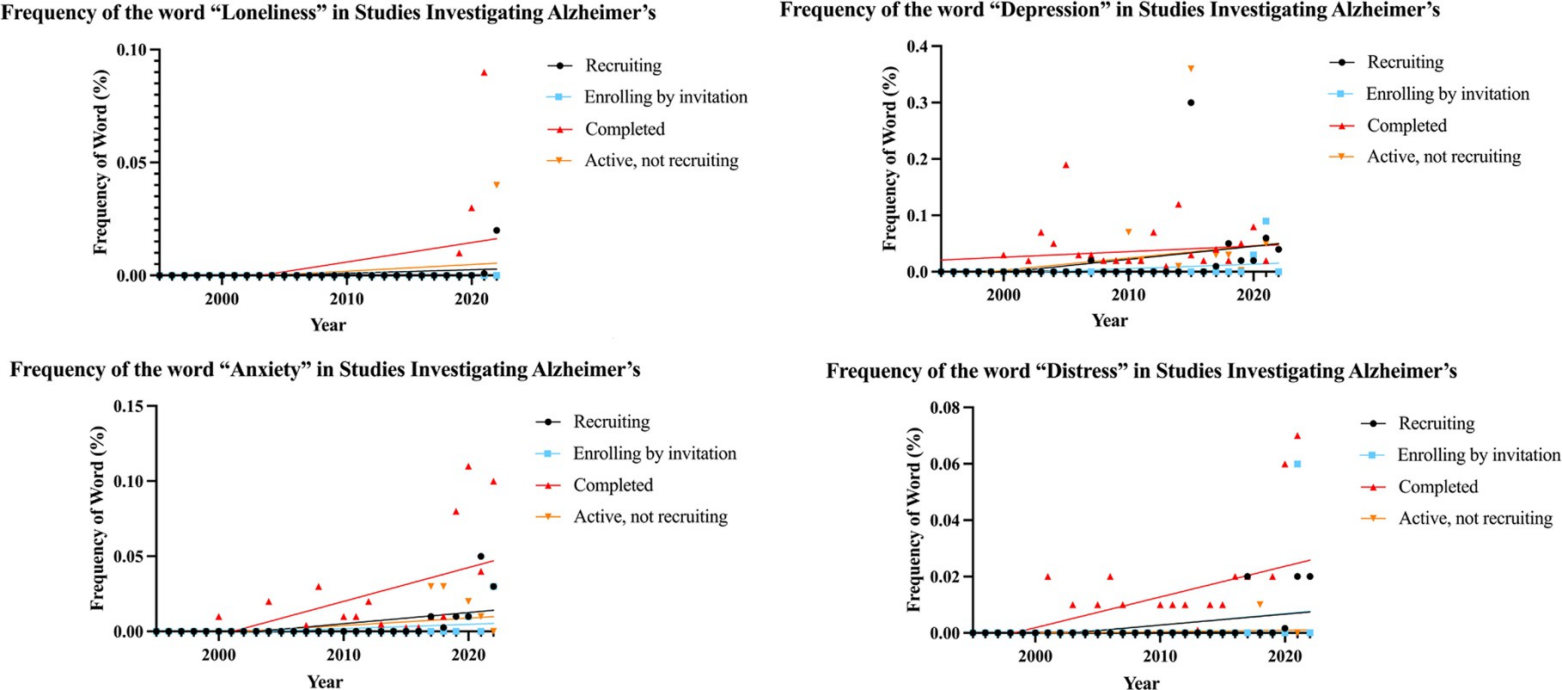
Association between the frequency of mental health terminology in the trial summary and the trial start year for loneliness (3a), depression (3b), anxiety (3c), and distress (3d).

We illustrated the frequency of the four MH terms in AD trial summaries by the trial start year in Fig 3. These terms only started appearing around the year 2000 and have demonstrated an increasing trend since, with a particular surge around 2020. Notably, the temporal growth was the most prominent for “anxiety” among completed trials (r = 0.61), while the correlation was the weakest (albeit still potentially important) for “depression” in completed trials (r = 0.20). In order of recruiting, enrolling by invitation, completed, and active, not recruiting studies, Pearson correlation coefficients were 0.335, 1.000, 0.400, and 0.322 for “loneliness”; 0.330, 0.373, 0.196, and 0.261 for “depression”; 0.557, 0.396, 0.613, and 0.467 for “anxiety”; and 0.508, 0.298, 0.526, and 0.226 for “distress.”

Trial COVID-19 period and completion status were not significantly associated with the odds of “depression” (Table 1). We conducted binary logistic regression analyses to assess the mention of each MH term over time (Table 2). Start year was significantly associated with the appearance of “anxiety,” “loneliness,” “distress,” and all words combined (Table 2). No significance was found for the mention of “depression” (Table 2).

**Table 2:** Binary logistic regression analysis on the effects of start year on the mention (y/n) of MH terminology (n = 2243).

	B	Standard Error	Wald	P-value	Exp(B)*	Lower 95% for Exp(B)	Upper 95% for (Exp(B)	Log Likelihood
Depression (y/n)	0.018	0.014	1.653	0.199	1.018	0.991	1.047	-568.041
Anxiety (y/n)	0.098	0.026	14.787	<0.001	1.103	1.049	1.160	-295.833
Loneliness (y/n)	0.554	0.186	8.838	0.003	1.740	1.208	2.507	-62.376
Distress (y/n)	0.052	0.026	3.972	0.046	1.054	1.001	1.110	-237.457
Any Word (y/n)	0.041	0.013	10.209	0.001	1.042	1.016	1.069	-691.823

*Exp(B) represents the odds ratio.

Kurtosis and skewness analyses were performed on the presence of the 4 MH terms (yes/no), finding that they were right-skewed and leptokurtic.

A multicollinearity analysis was run to assess interactions between the mention of any work and start year, COVID-19 period, and completion status. The variance inflation factors were 1.928, 1.921, and 1.825, respectively.

Sensitivity and specificity analysis on COVID-19 period and completion status were also performed (Table 3).

**Table 3:** Sensitivity and specificity analysis assessing the effects of COVID-19 and trial completion on the mention (y/n) of MH terminology (n = 2243).

	Depression (y/n)	Anxiety (y/n)	Loneliness (y/n)	Distress (y/n)	Any Word (y/n)
COVID? (y/n)					
Sensitivity (%)	27	43	83	38	32
Specificity (%)	76	77	76	76	77
Complete? (y/n)					
Sensitivity (%)	62	59	58	70	62
Specificity (%)	33	33	33	34	33

## Discussion

To the best of our knowledge, this is the first study that presents a program to extract and calculate the frequency of specific MH terms in AD trials. By applying this program to AD trials that are available on the U.S. National Library of Medicine ClinicalTrials.gov database, we found an increased presentation of three of the MH terms (loneliness, anxiety, and distress) over time, implying a consistently growing interest in incorporating these MH conditions by AD trial researchers.

Our results present several implications worthy of interpretation. Paramount among these is the increase in MH research interest, which we proxied using the frequency of depression, loneliness, anxiety, and distress in AD trial summaries over time. At face value, this might indicate that AD trial researchers started adopting an MH-focused approach around the turn of the millennium—an approach that has been growing since. This is contiguous with the literature, which demonstrates a consistent growth in MH awareness in AD research in the past two decades [21]. For instance, a time-trend analysis study in studies published before April 2011 found a significant rise in MH literacy among the public over two or more years [22]. Multiple papers have also examined the relationship between patient mental health and AD outcomes [23–25].

The multivariable analyses found significant associations between COVID-19 and the mention of “loneliness,” “distress,” “anxiety,” and the “any word” categories. Completion status was associated with “loneliness” and “distress” only. Odds ratios for these analyses varied greatly, with loneliness achieving a 46.810 for COVID-19 status. A potential association is the fact that isolation during the pandemic increased loneliness [26]. Isolation has been linked to numerous negative outcomes, with cognitive decline being of particular interest as that would be expected to increase a focus on loneliness with the advent of the pandemic [27]. Furthermore, the restricted life space we see in isolation, especially in high-risk environments like retirement homes, has been linked with an increased risk of AD—yet another reason to examine loneliness and themes of isolation in AD research [28]. On a more general note, the finding that COVID-19 status had a significant association in four out of the five MH categories analyzed is highly fascinating, demonstrating that in terms of significance, compared to completion status, COVID-19 status has a more consistent connection to the percent appearance of our MH terms (excluding “depression”). This might be explained by the global 25% increase in the prevalence of anxiety attributed to COVID-19 [29]. These findings suggest AD trial researchers have generally been quick to react to the potential surge in MH crises during the pandemic by incorporating MH conditions in their trials. Ultimately, there is a clear increase in MH term utilization. Since we did not examine the content of the trials beyond counting the appearance of MH terms from the summaries, we could not describe exactly how researchers operationalized them, which could hint more at the direction of AD research. This requires further study into the contexts of these trials.

The bulk of the time-trend analysis came from the binary logistic regressions that found significant positive associations between year and the appearance of MH terms (yes/no) for “anxiety,” “loneliness,” “distress,” and all terms combined. This suggests an increased emphasis placed on these terms over the years, with newer clinical trials choosing to cover these MH themes. The regression also had a low standard error for all the terms except “loneliness,” indicating a strong relationship for most models. Interestingly, the term “loneliness” had the highest odds ratio. The rise in single-person households may provide some explanation [30]. This increase in people living alone may be associated with feelings of loneliness among individuals. An increase in loneliness may be particularly striking in individuals within the risk/age group for developing AD as younger individuals may be moving to urban centers for career opportunities, leaving small towns with aging populations behind [31, 32]. Research performed in Finland found that aging and the increase in single-person households are associated with increased time alone [33]. These metrics all demonstrate an increase in loneliness, which may explain its increased time under the academic spotlight and the results of the binary logistic regression performed on our data.

The sensitivity and specificity analyses showed the COVID-19 period to be more specific and completion status more sensitive. This is not surprising as the natures of these two factors suit them to their respective outcomes. COVID-19 is an event that is intrinsically linked with time since it only started several years ago, meaning the studies that happened to mention these MH terms before the pandemic as a result of the generally growing trend in MH focus in AD research would be seen as false negatives. These would severely hurt the sensitivity score for COVID-19 as it is not the only factor behind the presence of MH terms in these clinical trials. On the other hand, specificity would not suffer as much as there are relatively few studies that mentioned the MH terms in this database. This means that specificity would naturally stay high as significantly fewer trials were present after COVID-19 than before. In other words, the high specificity of COVID-19 for the presence of MH terminology is unimportant. On the other hand, the sensitivity of completion status for MH terminology shows that it is more impactful. This is not to say that COVID-19 does not have an impact, as it still has some sensitivity and has been shown to influence the presence of MH terminology in these trials. It simply means that it has less of an effect than completion status. The collinearity analysis also shows that these effects do not overlap, demonstrating that both COVID-19 and completion status independently affect the outcome.

Notably, “depression” has not shown significance in any of these analyses, which may be attributed to the fact that it makes up a majority of the mentions (75%) and appeared earlier than most other terms, with several mentions in 2000. This is likely due to the fact that depression was diagnosed in AD patients earlier on, and thus, this database, which only provides consistent data in our format starting in 1995, is not old enough to find much of a trend. For example, a paper by Reifler & Eisdorfer published in 1980 diagnosed 18/82 of their Alzheimer’s patients with depression and noted that more patients met less stringent criteria [34]. If research making the connection between depression and AD is so much older than this database, this paper’s data are simply unsuitable for analyzing trends for the mention of the term.

In addition, studies have shown that an MH focus in AD treatment produces favorable outcomes [35]. In fact, psychological therapies have successfully improved outcomes for people with early to middle-stage dementia. These findings and the corresponding literature indicate that improving attitudes towards mental health treatment as a standard adjunct for AD patients may result in greater treatment completion. The growing trend found in completed trials for three of the four terms studied may indicate an association between the inclusion of MH terms and a trial being successfully carried out. The MH term that is the exception here is “depression,” which may indicate that these trials contain specific obstacles interfering with their completion. Perhaps target patients are less likely to adhere to the course of treatment or present another barrier to success for the trial. If nonadherence is the problem, there may be some value in determining how to improve adherence for depressed patients.

Ultimately, the goal of this proof-of-concept analysis was to develop a Python program for four MH conditions that could be later expanded to exhaustively capture all relevant MH terms in a trial summary page. This tool is an analytical method that can easily be extended to study other terms beyond the ones we used in the current analysis; as such, this study represents a tool prototype with potentially broad use. Furthermore, future studies can expand our method, preferably with more data points, by accessing larger datasets to perform more analyses.

### Limitations

Certain limitations exist in our analysis. The database is too young to examine whether there is a greater trend in the mention of “depression.” Furthermore, we did not attempt to understand the context of MH terms in the trial summary but rather considered any appearance to indicate a true intention of researchers to consider MH. We used the mention and frequency of MH terms as a simple proxy for research interest, although a word being frequently used does not necessarily indicate a more intensive examination. For instance, a trial with a shorter summary section will result in the MH term constituting a higher percentage of the text. Nevertheless, this is consistent with our purpose, as a shorter summary opting to include the MH term was likely more interested in evaluating the prevalence/impact of said term, compared to a longer description mentioning the term as an off-hand remark. Future research could expand our study to capture the entire sentence that contains these MH terms or isolate MH terms in the objective statement to characterize the context more accurately. They may also consider using a more objective measure, such as MH incorporation into the experimental protocol, to confirm our preliminary findings. Additional MH characteristics and behavioral symptoms that pertain to AD management, such as agitation and insomnia, are beyond the scope of this study.

## Conclusion

We developed a Python program to extract the frequency of four MH terms in AD clinical trials to show their temporal trends (and by proxy, research interest) in the past three decades. There was a statistically significant increase in the use of the terms loneliness, anxiety, and distress by year. This may indicate a heightened awareness among AD researchers of the need to incorporate MH variables in a trial setting. An increase in the mention of these three words was also observed when comparing studies before 2020, compared to those starting at or after 2020. Future studies are needed to delineate the context of MH terms to characterize the research interests more precisely. The Python program we developed represents a useful tool that may help inform future successful trials by easily examining trends in meaningful subjects.
